# Prevalence of Polycystic Ovarian Syndrome among Medical Students of a Tertiary Care Hospital

**DOI:** 10.31729/jnma.4885

**Published:** 2020-05

**Authors:** K.C. Shreeyanta, Rakesh Kumar Shah, Avilasha Singh, Astha Prasai, Birat Bhandari, Saman Aryal, Asmita Khatri, Meena Thapa

**Affiliations:** 1 Dirghayu Guru Hospital, Chabahil, Kathmandu, Nepal; 2 Gajuri Primary Health Centre, Dhading, Nepal; 3 Kathmandu Medical College, Sinamangal, Kathmandu, Nepal; 4 Deurali Primary Health Care Centre, Nuwakot, Nepal; 5 Om Hospital and Research Centre, Chabahil, Kathmandu, Nepal; 6 Ministry of Social Development, Hetauda, Makwanpur, Nepal; 7 Department of Obstetrics and Gynaecology, Kathmandu Medical College, Sinamangal, Kathmandu, Nepal

**Keywords:** *polycystic ovarian syndrome*, *medical students*, *Nepal*

## Abstract

**Introduction:**

Polycystic ovarian syndrome is considered to be one of the most common endocrine disorders among women of reproductive age. Characterized by a triad of androgen excess, anovulation, infertility, and obesity the disease can lead to several complications like infertility, endometrial carcinoma. This study aims to find out its prevalence among female medical undergraduates.

**Methods:**

A descriptive cross-sectional study was conducted among female undergraduate medical students in a tertiary care hospital from 1^st^ to 7^th^ February 2018. Ethical approval was taken from the Institutional Review Committee (reference number 10012018). The sample size was calculated. Systematic random sampling was done. Statistical Package for the Social Sciences version 20.0 was used. Point estimate at 95% Confidence Interval was calculated along with frequency and proportion for binary data.

**Results:**

Out of 381 participants, the prevalence of polycystic ovarian syndrome was found to be 35 (9.18%) at 95% Confidence Interval (6.28-12.08). Eighty (20.99%) participants were reported to have prolonged menses, 28 (7.34%) tended to grow dark, coarse hair, 79 (20.73%) reported being obese or overweight, and milky discharge from nipple was present in 4 (1.049%).

**Conclusions:**

The prevalence of polycystic ovarian syndrome was found to be similar to other studies conducted in similar settings. But still, it is a growing endocrinological problem in the females of the reproductive age group. Early screening is necessary to prevent lifelong complications.

## INTRODUCTION

Polycystic ovarian syndrome (PCOS) constitutes most cases of the endocrine disorder among females.^1^ With a triad of androgen excess, anovulation, infertility, and obesity; manifestations such as enlarged polycystic ovaries, secondary amenorrhea, hirsutism, and infertility are seen.^2^

PCOS is becoming a more prevalent disorder among women of reproductive age with lifelong complications.^3^ Incidence of PCOS is increasing rapidly due to changes in lifestyle and stress. Some of the women who develop cardiovascular disease (CVD), hypertension, endometrial cancer, and type II diabetes later in life appear to have suffered from PCOS in earlier years.^4^ Also, Nepal lacks accurate prevalence data of PCOS.^5^

Hence, this study was conducted with the aim of finding the prevalence of PCOS among medical students which included females of reproductive age group.

## METHODS

A descriptive cross-sectional study was conducted in Kathmandu Medical College and Teaching Hospital (KMCTH), Sinamangal, Kathmandu, Nepal. After taking the ethical clearance from the Institutional Review Committee of KMCTH with reference number 10012018, data was collected from female medical students (MBBS, BDS, and B.Sc. Nursing) from first to a final year studying at KMCTH from 1^st^ to 7^th^ February 2018. Those students that were already diagnosed with PCOS, thyroid disorder, or pituitary disorder were excluded. Those who did not give their consent were also excluded. The sample size was calculated using the formula,

n= Z^2^ × p × (1-p)/e^2^

= 1.96^2^ × 0.0913 × (1- 0.0913)/0.02^2^

= 0.3187/0.0004

= 796.75

= 797

Where,

n= required sample size
p= prevalence 9.13%, taken from the previous study^1^
e= margin of error, 2%
Z= 1.96 at 95% Confidence Interval

**Taking the finite population i.e. total female students of** Kathmandu Medical College (N)= 520

Adjusted sample size= n/[1+(n-1)/N]

= 797/[1+(797-1)/520]

= 314.92

= 315

Therefore, the calculated sample size was 315. Adding a non-response rate of 10%, the sample size was 346.5 i.e. 347.

A total of 381 students were taken in the study. Systematic random sampling was done. A self-administered closed-ended questionnaire was distributed. A clinical tool for diagnosis of PCOS by Pedersen SD, et al.^6^ was used for screening of PCOS. This questionnaire included questions regarding PCOS clinical symptoms, which is the clinical tool for the diagnosis. Positive scoring was given to prolonged menses, hirsutism (excess hair growth in three or more places in the upper lip, chin, breasts, chest, back, belly, upper arms, and upper thighs), and obesity now or sometime in the past. Milky discharge from nipple is kept for the exclusion of other endocrine abnormalities that mimics PCOS symptoms and hence if present gave a negative score. Finally, a cumulative score of two or more, indicates that the participant has clinical PCOS. Height and weight were recorded by standard procedures that are, the digital weighing scale for weight and stadiometer for height. Body Mass Index (BMI) was calculated.

Information bias, reporting bias, social desirability bias, and non-response bias were encountered. The collected data were coded and entered in IBM Statistical Package for the Social Sciences version 20.0.

## RESULTS

Out of the 381 female students included in the study, the prevalence of PCOS was found to be 35 (9.18%) at 95% Confidence Interval (6.28-12.08).

**Figure 1:**
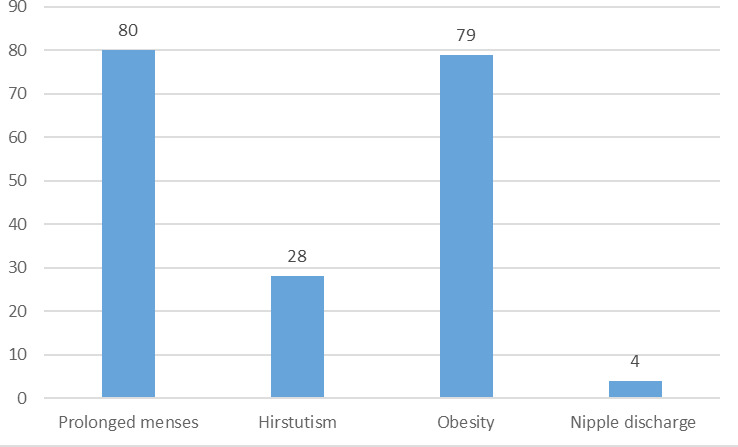
Clinical symptoms of PCOS.

Eighty (20.99%) participants reported that they had prolonged menses i.e. cycle more than 34 days or a totally variable cycle. 28 (7.34%) said they had the tendency to grow dark, coarse hair in three or more sites. 79 (20.73%) participants reported being obese or overweight now or sometime in the past. Milky discharge from nipple was present in four (1.049%) (Figure 1).

**Figure 2:**
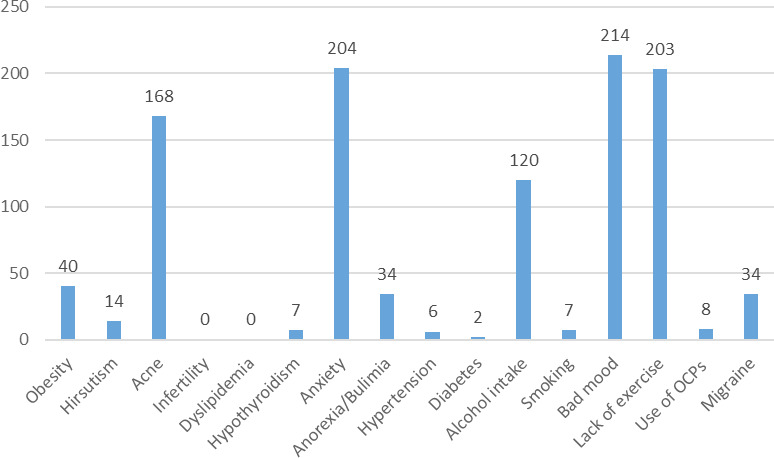
Presence of risk factors.

Among the 381 participants, 40 (10.5%) said they had obesity, 14 (3.7%) said they had hirsutism, 168 (44.1%) had acne, 7 (1.8%) had hypothyroidism, 204 (53.5%) had anxiety, 34 (8.9%) had anorexia/bulimia. Similarly, hypertension was reported in 6 (1.6%) participants, diabetes in 2 (0.5%), alcohol intake in 120 (31.49%), 7 (1.8%) smoked, and 214 (56.2%) had bad mood whereas 203 (53.3%) lacked physical exercise. Migraine was present in 34 (8.9%) and 8 (2.1%) used oral contraceptive pills (OCPs). None of the participants reported infertility and dyslipidemia (Figure 2).

**Figure 3:**
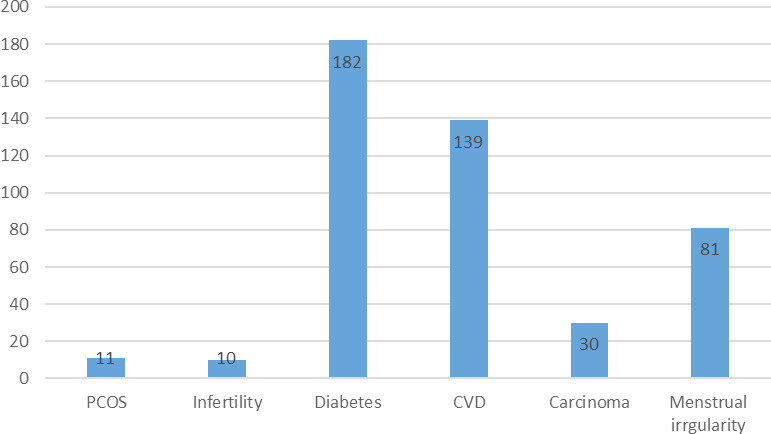
Family history.

Similarly, the family history of PCOS was present in 11 (2.9%), infertility in 10 (2.6%), diabetes in 182 (47.8%), CVD in 139 (36.5%), and carcinoma in 30 (7.9%). Eighty-one (21.3%) mothers of the participants had irregular menstruation (Figure 3).

## DISCUSSION

The worldwide prevalence of PCOS is estimated to be 6-7%.^7^ In our study, the prevalence was found to be 9.18% which is slightly higher than the worldwide prevalence.

In a study conducted in South India among health sciences, students the prevalence of PCOS was found to be 9.13%.^1^ Similarly, a hospital-based study done among Omani women showed the prevalence of PCOS to be 7%.^7^ Again, another study was done among young women in Bhopal, Central India which was found to be 8.2%.^8^ These findings were quiet similar to our findings.

Furthermore, the prevalence of probable PCOS was found to be high, 46.4% in a study done in Kashmir among reproductive age group women.^9^ Similarly, another study done among students of a tertiary care teaching hospital said that 32.11% met the criteria to be diagnosed as PCOS.^10^Our findings were considerably lower than these.

It is already known that PCOS has been attributed to several causes that include the change in lifestyle, diet, and stress.^4^ Studies suggest that epidemiological transition and rapid urbanization have led to the greater propensity of endocrine disorders among young adults in South Asia.^11^ In our study out of 381 participants, 204 (53.5%) reported having stress. Hence, stress was seen high compared to other risk factors in our participants also.

As our city is also subjected to rapid urbanization, dietary and lifestyle habits are also changing to unhealthy ones. In our study comparatively high number of participants reported alcohol intake 120 (31.49%) and lack of physical exercise 203 (53.3%). Such changes in lifestyle habits predispose adolescents and young women to diseases with long term complications due to insulin resistance like type II diabetes mellitus, PCOS.^1^ Another study adds even more attention to this problem saying that compared with other populations, South Asians have a greater propensity to insulin resistance and metabolic syndrome.^11^ In our study 40 (10.5%) reported to be obese currently or sometime in the past which was considerably lower than the study done in South India which reported 38.5% cases. Hence, education, self-empowerment, multidisciplinary care, and lifestyle intervention for prevention or management of excess weight are to be prioritized. Depressive and anxiety symptoms should be screened, assessed and managed.^12^

A study done in Nepal shows that the mean age of patients with PCOS was 24 years indicating that it is a disease mainly of young age.^5^This was again supported by another study done in PCOS which showed that out of 80 known participants with PCOS, 59 % of patients were in the age group of 20-29 years^6^ and it is clear that PCOS is a common disorder among young female.^1^ Hence, our study targeted this age group with an aim to screen and help in lifestyle modification to prevent lifelong complications.

The present study had certain limitations. It was conducted in only one medical college hence the findings cannot be generalized among all the female population. Since a descriptive cross-sectional study was conducted, the probable risk factors could only be quantified but a link between these probable risk factors and PCOS could not be established. The questionnaire has sensitivity 85.4% and specificity 93.4%.^7^ However, as only a clinical symptoms questionnaire was used in the study, it does not provide a probable diagnosis of PCOS. For more accurate diagnosis ultrasonography is recommended.

## CONCLUSIONS

The prevalence of PCOS was found to be similar to the previous studies done in a similar setting.

PCOS is becoming one of the most common endocrinological problems. Hence, early screening, lifestyle modification, and intervention are necessary to prevent lifelong complications. There is a paucity of research providing accurate data on the prevalence of PCOS in our country. Another study also suggests that the study of PCOS on a large scale would be helpful to provide a more accurate picture of the disease. Hopefully, this study will help identify the proportion of PCOD cases so that they can be subjected to further diagnostic tests, and thus apply lifestyle modification and treatment as early as possible to prevent lifelong complications. This a study involving a larger area and a larger sample size is recommended.

## Conflicts of Interest:

**None.**
